# Lipase-catalysed acylation of starch and determination of the degree of substitution by methanolysis and GC

**DOI:** 10.1186/1472-6750-10-82

**Published:** 2010-11-29

**Authors:** Apostolos Alissandratos, Nina Baudendistel, Sabine L Flitsch, Bernhard Hauer, Peter J Halling

**Affiliations:** 1 WestCHEM, Department of Pure and Applied Chemistry, University of Strathclyde, Thomas Graham Building, 295 Cathedral Street, Glasgow, G1 1XL, UK; 2 BASF-AG, Fine Chemicals and Biocatalysis Research, 67056, Ludwigshafen, Germany; 3 School of Chemistry, Manchester Interdisciplinary Biocentre, 131 Princess Street, Manchester, M1 7DN, UK

## Abstract

**Background:**

Natural polysaccharides such as starch are becoming increasingly interesting as renewable starting materials for the synthesis of biodegradable polymers using chemical or enzymatic methods. Given the complexity of polysaccharides, the analysis of reaction products is challenging.

**Results:**

Esterification of starch with fatty acids has traditionally been monitored by saponification and back-titration, but in our experience this method is unreliable. Here we report a novel GC-based method for the fast and reliable quantitative determination of esterification. The method was used to monitor the enzymatic esterification of different starches with decanoic acid, using lipase from *Thermomyces lanuginosus*. The reaction showed a pronounced optimal water content of 1.25 mL per g starch, where a degree of substitution (DS) of 0.018 was obtained. Incomplete gelatinization probably accounts for lower conversion with less water.

**Conclusions:**

Lipase-catalysed esterification of starch is feasible in aqueous gel systems, but attention to analytical methods is important to obtain correct DS values.

## Background

Naturally abundant polysaccharides, in particular starch and cellulose, are increasingly being considered as renewable and potentially biodegradable starting materials in many traditional industries [1,2]. In many applications, the properties of natural polysaccharides are not adequate for their intended uses and physical or chemical modification is employed in order to obtain a material with improved properties. It has been found that even small changes in the structure of the polysaccharide can lead to extensive alterations of chemical and physical properties [1]. Chemical modification usually aims to hydrolyse glucoside links in the chain, or to attach different types of molecules on to the chains, taking advantage of the three hydroxyls present in each anhydroglucose unit. This has led modified starches to become important commercial products, with a range of uses most notably in the food industry [2].

Biocatalysis is an attractive option for chemical modification of starch, and has been extensively used for partial hydrolysis to generate oligomers. However, the enormous range and diversity of available enzymes offers the possibility to obtain completely novel products with new or improved functionalities that were impossible to produce through traditional means. The use of enzymes to acylate the starch molecule, and generate compounds with commercial importance, is a relatively unexplored field [3-7]. Even where the modifications have been made previously, enzymatic routes may be preferable because the chemical processes include extreme pH conditions, solvents that push the limits of acceptability for health and other reasons, and substrates such as anhydrides and acid chlorides [2]. New possible uses of modified starches in fields such as the pharmaceutical or biomedical industries and even traditional uses are challenged by new waves of strict health and safety laws and regulations. This results in the need to update or replace traditional methods of modified starch formation with "cleaner" methods. Biocatalysis is a possible solution considered in situations where milder reaction conditions are required, with fewer by-products and higher selectivity.

Starch fatty acid esters have traditionally been dominated by starch acetates which presented more desirable properties for the intended uses. In recent years however starch acylates of medium or longer chain acids have emerged as candidates for novel uses, mainly in the biodegradable and renewable materials industries. One of the main advantages of these materials is the ability of longer chain esters to work as internal plasticizers of starch's glucan matrix [8]. The extent of acylation is characterised by the degree of substitution (DS), defined as the average number of acyl groups per anhydroglucose unit.

**Figure 1:**
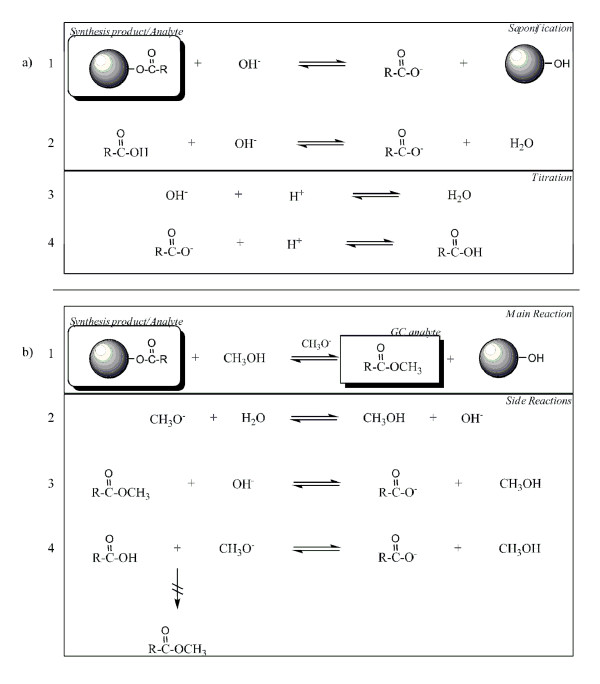
**Reactions and side-reactions of starch ester analyses**. a) Titration Analysis: 1 Hydrolysis of starch ester (circle denotes starch), 2 Saponification of acid, 3 Titration of residual hydroxide ions, 4 Titration of saponified acid, b) Transesterification/GC Analysis: 1 Methanolysis of starch ester and formation of methyl ester, 2 Hydrolysis of methoxide 3 Hydrolysis of methyl ester, 4 Deprotonation of acid to form acyl ion, no methyl ester synthesis.

When starch has been esterified, whether by conventional chemical or enzymatic routes, product analysis is important. The analysis of esterified starch samples has been approached in a few different ways. The oldest and most common method however has been a titrimetric one, first proposed by Genung and Mallatt [9]. The principle of the method is that if modified starch is saponified with a known amount of hot aqueous NaOH, the ester bonds will be hydrolysed and sodium acylates will form. When this solution is back-titrated with a standard strong acid (e.g. HCl), the amount of NaOH used for saponification can be calculated and consequently the acyl group substitution can be quantified (Figure 1a). This method is still widely employed, as shown by recent references [6,7,10-13]. Recently this method has also been used to quantify starch esters of fatty acids [6,7,11]. The case of cellulose has been approached with the same methodology. Saponification followed by titration has been used to a great extent, even in recent years [14].

Other more recent methods include the quantification based on the absorption band assigned to the vibration of the carbonyl in the ester group, with FTIR [15] and a method based on NMR spectroscopy [16]. However due to the need for instrumentation and the uncertainty of analysis of polymers with these methods, the titration analysis has been the method of choice in the past years. In recent years and with the introduction of specialised high throughput titration tools, there has been, to our knowledge, no attempt at a more insightful look at the parameters of this method. We attempted to perform this analytical approach, with an automatic titrator, which allows for precise readings and accurate titration of samples of small volume. These experiments revealed flaws that led us to seek another approach for an easy benchtop analytical method. Alkaline methanolysis is used in many cases to produce methyl esters via a transesterification reaction. The products are usually suitable for GC analysis, especially in the case of esters of medium chain length fatty acids. Methanolysis of cellulose acetates has been reported, followed by GC analysis of the methyl acetate [17]. Here we propose an analysis scheme for starch esters of fatty acids, based on treatment of the starch ester with sodium methoxide, followed by GC analysis of the resulting fatty acid methyl ester.

## Results and Discussion

### Potentiometric titration analysis

**Figure 2:**
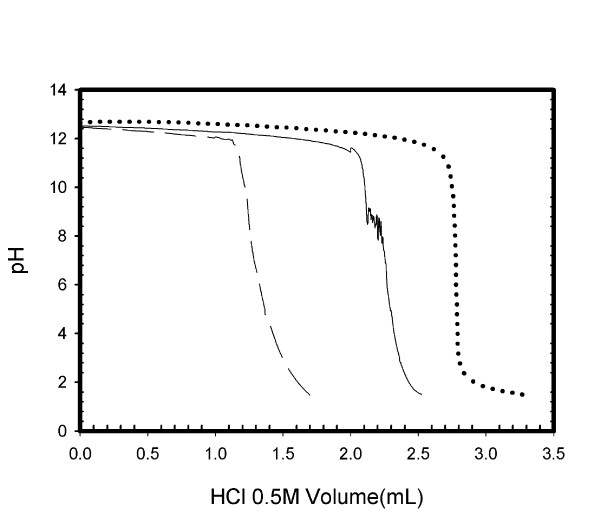
**pH curves from titration analysis**. Curves from titration by 0.5 M HCl of (···):1.4 mL of 1.0 M NaOH, (—): Native starch treated with 1.4 mL 1.0 M NaOH, (---): Native starch and decanoic acid mixture treated with 1.4 mL 1.0 M NaOH.

The published saponification method (Figure 1a) indicates that back-titration should end at the initial pH of the starch suspension (before NaOH addition), but does not specify a precise value. It is clearly important that the end-point pH value is sufficiently high that sodium acylate does not significantly titrate. To investigate the possible application to decanoyl starch, we ran some standards and controls on an automatic titrator. These, presented in Figure 2 indicated some issues that compromise the method's reliability.

Figure 2 shows the potentiometric titration curves obtained after the saponification treatment was applied to native starch and its mixture with decanoic acid. There are a few points to makes on the resulting curves. It should be noted that the viscosity of the solution is high, as might be expected (alkali induced gelatinisation), making it difficult to homogenise the solution during titration. This may cause localized higher or lower pH values, and probably accounts for the noisy artifact visible on the titration curve of the starch blank, which was typically observed. However, the starch blank curve is clearly not close to the sodium hydroxide curve. There seems to be a generation of acid during the saponification treatment of starch, which neutralizes some of the sodium hydroxide. This could be caused to a certain extent by a slow alkaline decomposition and partial oxidation by atmospheric oxygen, which generates small carboxylic acids, such as ethanoic and lactic acid, as known to occur during the alkaline treatment of starch [9,18]. This means that it is necessary to take into account this contribution when quantifying a modified product. From these results it is clearly necessary always to use a titration of the native unmodified starch, as a blank. This blank is only mentioned in a few early publications [19]. It might be considered obvious that a blank should be used, but it is possible that some workers feel that the blank will be negligible, as native starch does not contain significant numbers of acid or basic groups. However, even if a blank is used, in our experiments these starch blanks were found not to be very reproducible, nor to vary proportionally to the amount of starch used. Together with the fact that the starch blank can be a very large correction, this makes the method not reliable.

Another problem is the nature of the titration curve for the starch/fatty acid mixture. When sodium decanoate was titrated alone, there was a clear phase of significant acid consumption between pH of around 8 to 6. This titration occurs well above the true pK value, because the neutral decanoic acid formed comes out of solution, pulling the equilibrium over. However, after the saponification treatment in a mixture with starch, there is no indication of this titration of sodium decanoate (Figure 2). The curve does show the expected consumption of NaOH by initial neutralization of the decanoic acid. This means that even though a portion of NaOH neutralizes the decanoic acid, the subsequent titration of the sodium decanoate can not be clearly recorded. We also found the pH of the aqueous starch mixture, prior to sodium hydroxide addition, to have values close to 6. This of course means that when back titrating in order to reach a pH of 6, some of the sodium decanoate molecules formed would also be titrated, thus adding to the errors in the DS quantification.

These same problems were found when using the titration method with a range of different starch types (data not shown). Overall, our observations suggest that the titration method is unreliable, at least for starch esters of medium chain fatty acids. It might be acceptable for high DS acetyl derivatives, for example, where blanks may be smaller relative to the sample titrations.

### Transesterification/GC analysis

Transesterification of acetyl groups from starch to methanol has been employed, where the resulting methyl acetate was distilled, then analysed with alkaline saponification and titration [20,21]. In the case of longer chain fatty acids (Figure 1b), the resulting fatty acid methyl ester (FAME) can be easily detectable with great accuracy on a gas chromatography system. We therefore investigated GC detection of FAME as an alternative to the titration method described above. At first, the GC analysis was optimized for speed, accuracy and reproducibility. Initial problems with irreproducibility due to heterogeneity of the solid starch product were overcome by dissolution of the whole reaction product in DMSO until a clear solution was obtained (20-50 mg/mL DMSO).

In initial trials the quantity of methyl ester found declined again after longer transesterification times, probably because of its hydrolysis. The rate of disappearance was faster at higher temperatures, but could be reduced by the use of special grade anhydrous methanol. A transesterification reaction at 50°C gave maximum methyl ester at just under one hour. The hydrolysis of the methyl ester was not detectable under these conditions.

Once the FAME was quantified, the average mol of acyl groups per anhydroglucose unit was calculated to give the DS of the modified starch. As there are three hydroxyl groups per unit present, the maximum value the DS can reach is 3. It should be pointed out that when used for systems with fatty acid substrates, any residual free fatty acid in the final product will not esterify and thus will not affect the resulting DS. This is expected for alkaline methanolysis, because the fatty acid is converted to the unreactive carboxylate anion. Nevertheless, because we were measuring small DS values, a control was performed where decanoic acid was added to the mixture prior to transesterification. No detectable methyl ester was found, unless fatty acid esters were also present. This is a very important advantage of this method, as, in our experience, it is not uncommon for unreacted fatty acid to remain non-covalently attached to the starch, even after several washes. The titration analysis, will not distinguish between covalently and non-covalently bound residues.

The reproducibility of the method was tested by replicate analysis of samples taken from the same reaction product, showing a standard deviation of less than 3% (e.g. DS of 0.0042 ± 0.0001).

### Lipase catalysed esterification in concentrated aqueous gelatinised starch systems

**Figure 3:**
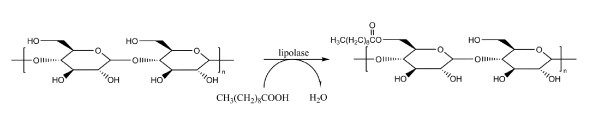
**Enzymatic acylation of starch**. With lipolase, at 50°C for 60 minutes, mixed by overhead stirrer.

Systems using lipase-catalysed esterification of gelatinized starch with a low water concentration, surfactant and a free fatty acid have been described in literature [6,7] (Figure 3). In our hands, these systems show rather low homogeneity and ability to be mixed.

The analysis of these enzymatic reaction systems with the GC method described earlier showed clear peaks of methyl decanoate, whilst controls (no enzyme) gave very small peaks, or no peaks at all. Quantification of these peaks showed that starch decanoate with a DS ranging from 0.004-0.007 was obtained (Table 1). Such DS values are close to 0.01, which is said to be approximately the target for most starch acylates with desirable properties, as further acylation alters too much the physical characteristics of starch [1,22,23]. Furthermore we found the presence of surfactant unnecessary for the reaction to proceed, as similar DS were recorded for systems with or without added Triton X-100. Presumably the viscous nature of the aqueous phase is enough to ensure adequate dispersion of the fatty acid. It should be noted that the product of these reactions did not appear to be homogeneous, and could be roughly separated into two fractions. One fraction retained the powdery appearance of native starch, while the other fraction was large particles of aggregated starch, possibly containing some other reaction ingredients that could not be removed by washing. This fractionation was more profound for higher amylose contents. The existence of these fractions made the dissolution of the whole product in DMSO prior to analysis even more important. Only in this way could a representative sample be taken in order to obtain reproducible and accurate measurements of bulk acylation.

**Table 1:** Degrees of substitution for enzymatic acylation of different starches with decanoic acid using lipase from Thermomyces lanuginosus (lipolase)

Starch source	DS^a^	% Amylose
Amylose	0.0025 ± 0.0006	100%

Hylon VII (high amylose)	0.0039 ± 0.0005	70%

Wheat	0.0042 ± 0.0001	25%

Maize	0.0040 ± 0.0006	25%

Tapioca	0.0093 ± 0.0011	17%

Amioca (high amylopectin)	0.0084 ± 0.0010	< 1%

^a^Analysis with transesterification/GC method, errors are standard deviation for triplicate experiments, all controls had DS < 0.00001. The values for amylose content were given by the suppliers of each starch type.

Another point is that in general, starches with higher amylopectin fractions seem to result in higher DS values (Table 1). Rather than the chemistry of the molecule itself, this probably can be related to the effect of amylopectin content on the rheology of the gelatinized starch, which likely influences the mixing of the system.

It is worth noting that a control where the enzymatic reaction mixture is analysed without incubation (yield for time zero), did not result in the generation of methyl ester. This control was run in case any methyl ester was generated from residual fatty acid, catalysed by residual lipase, during the work-up and analysis process.

**Figure 4:**
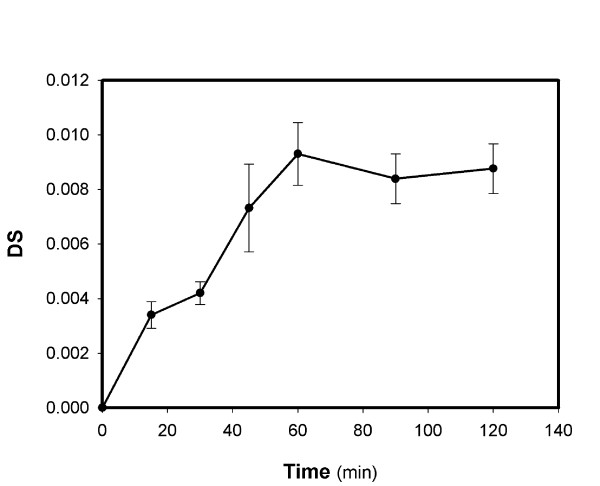
**Progress of lipolase catalysed synthesis of tapioca starch decanoate**. This used standard conditions with 1.5 mL H_2_O per g starch during gelatinisation.

The reaction progress with time was monitored for the starch type that resulted in the higher DS. From Figure 4 it is obvious that the reaction reaches a plateau after one hour, though whether it is a true equilibrium can not be deduced from these data alone.

**Figure 5:**
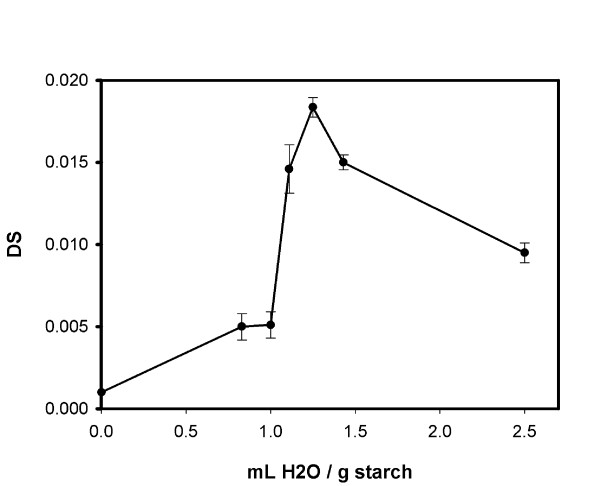
**Change in obtained DS for different initial concentrations of tapioca starch/water mixtures**. This shows the ratio of starch to water mixed prior to the gelatinisation stage. Water content of the starch, and water losses were negligible, but additional water is present during the enzyme reaction, because this is added as an aqueous solution (0.5 mL per g starch). Reaction time was 1 hr.

Because water plays an important role in both stages of the process, as a gelatinization medium and as an enzymatic reaction solvent, the effect of water concentration in the gelatinization mixture was also investigated. It seems that although the enzymatic reaction as expected is favored by reduced water concentration (or increased starch concentration), a minimum amount of water is necessary for the progression of the gelatinization. This seems like a reasonable interpretation of the optimum observed in Figure 5, which displays this trade-off between increased enzymatic synthetic activity at lower water concentrations and complete disruption of the granular structure at higher water concentrations.

### Product analysis by NMR

**Figure 6:**
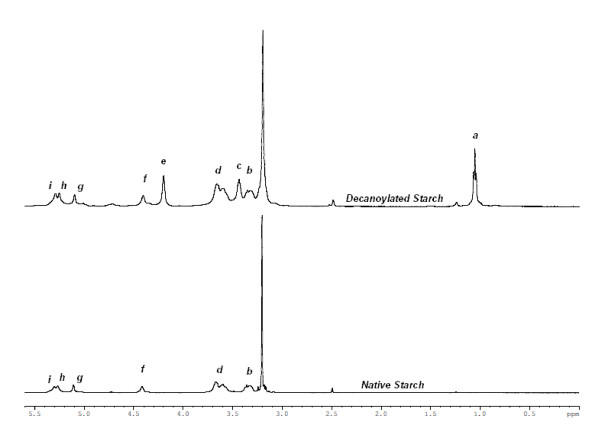
**NMR Spectrum for starch decanoic acid ester and unmodified native starch**. The large peak at 3.2 ppm can be attributed to water from the starch sample.

We attempted to analyse the product of an enzymatic starch acylation by nuclear magnetic resonance and compare the spectrum with that of unmodified native starch. With the aid of a COSY experiment, we successfully assigned the peaks corresponding to carbohydrate protons. Figure 6 shows the NMR spectrum for decanoylated starch ester. Peaks b (3.322-3.357 ppm), d (3.601-3.668 ppm) and g (5.108 ppm) can be assigned to the CH protons from the C4/C2, C5/C3/C6 and C1 anhydroglucose carbons respectively. Peaks f (4.415 ppm), h (5.264 ppm) and i (5.305 ppm) are due to protons from the hydroxyl groups of C6, C3 and C2 carbons respectively. As well as these peaks assignable to the carbohydrate, we also found three extra peaks, appearing exclusively in the modified starch spectrum, a (1.006-1.072 ppm), c (3.446 ppm) and e (4.207 ppm), and not recorded with native starch. The upfield peak was at a shift that has been attributed to protons in the fatty acid chain in previous published work on starch fatty acid esters [3-5]. A scan of the same sample after addition of deuterated methanol verified our peak assignments for the starch protons, as the hydroxyl proton peaks disappeared, but also resulted in the loss of peak e. This made us suspect the possibility of residual ethanol from the washing stages of the reaction. Our suspicions were verified by measurement of the spectrum of ethanol in the same DMSO-d_6 _medium, showing perfect agreement of chemical shifts with peaks a, c and e. We further analyzed a sample of acylated starch precipitated with acetone instead of ethanol. The peaks attributed to ethanol were indeed now missing, with instead a large peak appearing at 2.08 ppm, as expected for acetone protons. Small low-shift peaks were revealed, which might be attributable to methyl and methylene protons in the decanoyl chains. Their small size made accurate quantitation difficult, but estimated DS values were about twice those from the GC method. However, fatty acid non-covalently attached to the carbohydrate would also contribute to these peaks. No ^1^H-NMR peaks were detectable that could be assigned exclusively to covalently (or non-covalently) bound fatty acid. Hence we conclude that this NMR approach is not a reliable method for estimation of DS in the low range encountered with our samples. It may well be accurate for much higher DS starch derivatives, especially when purified (one report [24] suggests that the method should be used for DS > 2). On the contrary, as mentioned above, the transesterification/GC analysis does not have a contribution from non-covalently attached fatty acid, and can accurately measure low DS values.

## Conclusions

We have developed a new analysis scheme for determining degrees of substitution in starches acylated with fatty acids. This analysis method has been used to evaluate a number of enzymatic acylation reactions and to measure the DS for a number of acylated starch types. We found an optimum water concentration in the gelatinization mixture, which can be attributed to its role in the disruption of starch's granular structure while inhibiting the synthetic reaction. Thus, acylation of Tapioca derived starch using lipase from *Thermomyces lanuginosus *(lipolase) with decanoate was achieved to substitution yields of close to 0.02 DS with good reproducibility.

## Methods

### Enzymatic esterification of starch

Hylon VII (70% amylose), Tapioca Starch and Amioca Powder TF (near 100% amylopectin) were provided by National Starch (Germany). Amylose, lipase from *Thermomyces lanuginosus *(lipolase), Triton X-100 and decanoic acid were obtained from Sigma-Aldrich(UK).

Starch (dried in a convection oven to constant weight) was mixed with sodium phosphate buffer (0.1 M, pH = 7.0), at a ratio of typically 40% w/v in a 50 mL round bottomed flask and gelatinised while mixed at 90°C, for approximately 1 hour, followed by cooling to 50°C. 0.5 mL of lipolase solution (lipase from *T. lanuginosus*, approximately 50 kU for Sigma assay), per gram of starch were added and mixed thoroughly. Finally 0.5 g of liquid decanoic acid per gram of starch (0.46 mol per mol anhydroglucose units) was placed in the vessel. The reaction solution was mixed with an overhead stirrer (Heidolph, anchor paddle, 50 rpm), for 1 hour. Smaller scale reactions were carried out in 2 mL screw-top centrifuge tubes and mixed in a Vortex Disruptor Genie. The reaction solution was then quenched with 10 times its volume of acetone or absolute ethanol, to precipitate the starch. Ethanol is most commonly used in published methods to precipitate starch, but we found that its use could lead to formation of small but detectable amounts of ethyl decanoate, probably through catalysis by the enzyme still present. The amounts found (by GC analysis) corresponded to less than 0.0005 DS, but it is recommended to avoid ethanol, especially when low DS values are to be measured. After centrifugation (4000 rpm or 2690 g) the supernatant was removed and the product was washed twice with the same volume of acetone or ethanol. After washing, the product was dried in a desiccator under vacuum. A control used an equal volume of buffer instead of enzyme solution.

### Potentiometric titration analysis

Potentiometric titration analysis was performed in an automatic titrator (Radiometer Analytical, TIM 854). The substituted starch (300 mg) was treated with H_2_O (3.6 mL) at 30°C for one hour, followed by measuring the pH, and further treating with 1.4 mL 1.0 M NaOH at 50°C for 48 hours [10]. The solution was then titrated with a volumetric 0.5 M HCl solution until the pH reached a value of approximately 1.5, so the full titration curve could be observed. The literature method would suggest DS is calculated from the amount of NaOH consumed during the saponification, after the excess has been titrated back to the pH value measured prior to NaOH addition.

### Transesterification/GC analysis

Anhydrous methanol and sodium methoxide in methanol solution were obtained from Sigma-Aldrich (UK). A small sample (5-30 mg) of esterified starch dissolved in 0.5 mL DMSO was mixed with 1 mL of sodium methoxide 0.07 M in methanol solution and a known amount (10 μL of a 100 mg/mL solution in n-heptane) of internal standard (n-tetradecane was suitable in the case of decanoic acid). Reagents were of anhydrous grade to minimise hydrolysis of the methyl ester as a side-reaction. This mixture was then heated (50°C) under reflux for 60 min, while shaken, then cooled and 1 mL of deionised water and 1 mL of n-heptane were added. The mixture was shaken for 1 min and left to settle. The top organic phase contained the methyl ester and could be removed and injected into the GC-FID (Perkin-Elmer Autosystem XL with a CP Simdist capillary column, oven set at 120°C, the injector at 130°C and the detector at 150°C).

### NMR analysis

^1^H and 2D-COSY experiments were conducted at 50°C on an AVANCE/DRX500 spectrometer at 500 MHz. The starch samples were dissolved at 50°C in DMSO-d_6 _or DMSO-d_6 _: methanol-d_4 _(4:1) at a concentration of 10 mg/mL (DMSO-d_6 _and methanol-d_4 _were obtained from Sigma-Aldrich). The samples were incubated at 50°C for 5 min in the spectrometer, prior to analysis.

## Authors' contributions

AA performed all experiments, participated in all data analysis, and drafted much of the manuscript. NB and BH provided regular advice as the study progressed. SLF and PJH conceived of the study, participated in all data analysis, and drafted parts of the manuscript. All authors read and approved the final manuscript.
